# Draft Genome Sequence of *Rhizobium* sp. Strain AG207R, a Potential Bacteriocin Producer Isolated from Ginger Roots and Exhibiting Broad-Spectrum Antimicrobial Activity, Including against Multidrug-Resistant Enterococcus faecalis

**DOI:** 10.1128/mra.00055-23

**Published:** 2023-05-24

**Authors:** Wongsakorn Kwandee, Sirirat Boondireke, Wuttichai Mhuantong, Darika Kongrit

**Affiliations:** a Faculty of Science and Industrial Technology, Prince of Songkla University (Surat Thani campus), Surat Thani, Thailand; b Department of Stomatology, Faculty of Dentistry, Srinakharinwirot University, Bangkok, Thailand; c Enzyme Technology Research Team, National Center for Genetic Engineering and Biotechnology, Pathum Thani, Thailand; d Faculty of Innovative Agriculture and Fishery Establishment Project, Prince of Songkla University (Surat Thani campus), Surat Thani, Thailand; University of Arizona

## Abstract

Here, we report the genome sequence of *Rhizobium* sp. strain AG207R, isolated from ginger roots. The genome assembly comprises a 6,915,576-bp circular chromosome with 59.56% GC content and possesses 11 regions of biosynthetic gene clusters of secondary metabolites, including one related to bacteriocin.

## ANNOUNCEMENT

**FIG 1:**
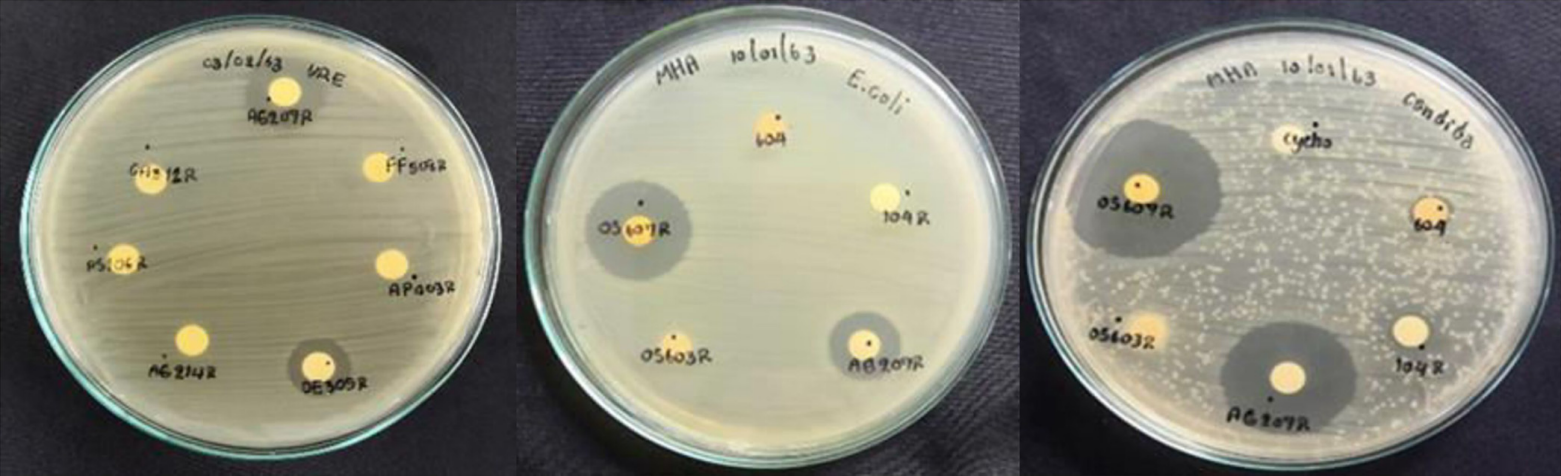
Preliminary screening of antimicrobial activities of the crude extracts of AG207R and other rifampin-resistant isolates against Gram-positive bacteria (vancomycin-resistant Enterococcus faecalis ATCC S1299 [VRE]) (left), Gram-negative bacteria (Escherichia coli) (center), and nonfilamentous fungi (Candida albicans) (right). Crude extracts of all isolates were prepared using butanol as an organic solvent, evaporated, and dissolved in dimethyl sulfoxide. A concentration of 5 mg of AG207R crude extract in 25 μl was dropped on the sterile filter paper disc (6 mm in diameter). All the test strains were grown in Mueller-Hinton broth (Himedia, India) at 37°C for 24 h and adjusted to 10^8^ CFU/mL, corresponding to a 0.5 McFarland standard (22).

Bacteriocins are antimicrobial peptides that are produced by bacteria to inhibit the growth of other bacteria, including closely related species (1–6). The majority of genes encoding bacteriocins are located in plasmids (6–9); however, certain genes are from chromosomes (9–12). We have isolated *Rhizobium* sp. strain AG207R from ginger roots. The strain was found to exhibit antimicrobial activity against a range of test pathogens, as shown in Fig. 1. To gain a better understanding of the biosynthetic gene clusters (BGCs) encoding secondary metabolites, particularly those related to antimicrobials, the whole-genome sequence of AG207R was investigated.

The strain was isolated from roots of ginger growing in Surat Thani, Thailand (8°44′29.8″N, 99°00′07.0″E). We applied antibiotic resistance-mediated isolation (13) to screen for the potential endophytic bacterial producers isolated from herbaceous roots. Rifampin was used as a screening filter to isolate novel antimicrobial ansamycin-specific scaffolds from actinomycetes, which originally were our focus as they have been major sources of bioactive substances; however, after performing preliminary screening to test for antimicrobial activities from the acquired rifampin-resistant isolates using a disc diffusion assay, we found *Rhizobium* sp. AG207R with promising antimicrobial activity. The strain was grown on Bennett’s agar (13), preserved in 25% glycerol, and kept at −80°C. It has been deposited at NITE Biological Research Center (NBRC) and Thailand Bioresource Research Center (TBRC) under accession numbers 115229 and 13869, respectively.

A single colony of AG207R was inoculated in yeast mannitol medium and grown at 30°C with shaking at 200 rpm for 3 days. Genomic DNA was prepared using the GF-1 bacterial DNA extraction kit (Vivantis, Malaysia). All procedures were performed following the manufacturer’s instructions. The NEBNext Ultra II DNA library prep kit (New England Biolabs [NEB], MA, USA) was used for the library preparation that was performed with standard parameters.

Genome sequencing was performed on the Illumina HiSeq platform (paired end [PE], 150 bp), resulting in 4,852,755 pairs of raw reads that were initially cleaned by trimming adapter sequences and removing low-quality sequences (Q score of <20) using Fastp version 0.20.0 (14). The cleaned sequences were assembled using SPAdes version 3.10.1 (15), with a minimum contig size of 500 bp. The contiguity of assembled sequences was evaluated by using QUAST version 5.0.1 (16). The gene completeness of genome assembly was assessed by using BUSCO version 5.4.2 (17), using the “bacteria_odb10” in the OrthoDB database (18). Genome annotation was performed using Rapid Annotation using Subsystem Technology (RAST) version 2.0 (19). BGCs of AG207R were predicted by antiSMASH version 6.0 (20) and ARTS (Antibiotic Resistant Target Seeker) version 2 (21). antiSMASH was implemented using the default options, “loose” detection strictness, and “All on” extra features. ARTS was implemented using the default options with “Alpha-Proteobacteria” for “Reference set.”

The genome size of AG207R was 6,915,576 bp, comprising 37 contigs with 210× genome coverage. The longest contig size was 877,345 bp, with an *N*_50_ value of 468,311 bp. The GC content was 59.56%. RAST genome annotation identified 6,967 genes, including 6,912 protein-coding genes, 52 tRNA genes, and 3 rRNA genes. All genome assembly and annotation statistics are summarized in Table 1.

**TABLE 1:** Genome sequencing statistics

Parameter	Value
Raw reads	
No. of reads (PE)	4,852,755
Coverage	210×
Genome assembly	
Total length (bp)	6,915,576
No. of total contigs	37
No. of contigs of ≥10,000 bp	24
No. of contigs of ≥50,000 bp	21
Largest contig (bp)	877,345
*N*_50_	468,311
*L*_50_	6
GC (%)	59.56
No. of N’s	393
No. of N’s/100 kbp	5.68
Genome assembly assessment	
% complete BUSCOs (C)	100%
% complete and single-copy BUSCOs (S)	98.40%
% complete and duplicated BUSCOs (D)	1.60%
Genome annotation	
No. of predicted genes	6,967
No. of protein-coding genes	6,912
No. of tRNAs	52
No. of 5S rRNAs	1
No. of 16S rRNAs	1
No. of 23S rRNAs	1

Analyses using both antiSMASH and ARTS platforms unveiled secondary metabolite BGCs in 11 regions, including one bacteriocin, one nonribosomal peptide synthetase (NRPS), one NRPS-like BGC, two polyketide synthases (PKS), one terpene, and one hybrid BGC (NRPS-T1PKS).

### Data availability.

This whole-genome shotgun project has been deposited in GenBank under the accession number JAEPVT000000000. The version described in this paper is the first version, JAEPVT010000000. The data are available under NBCI BioProject accession number PRJNA690836. Raw reads have been deposited in the NCBI SRA under accession number SRR22540942.
